# Hypocholesterolemic Effects of Lactic Acid-Fermented Soymilk on Rats Fed a High Cholesterol Diet

**DOI:** 10.3390/nu4091304

**Published:** 2012-09-18

**Authors:** Maki Kobayashi, Rie Hirahata, Shintaro Egusa, Mitsuru Fukuda

**Affiliations:** 1 Department of Food Science and Nutrition, School of Human Environmental Sciences, Mukogawa Women’s University, 6-46 Ikebiraki-cho, Nishinomiya, Hyogo 663-8558, Japan; Email: maki1002@mukogawa-u.ac.jp; 2 Food Science and Nutrition Major, Graduate School of Human Environmental Sciences, Mukogawa Women’s University, 6-46 Ikebiraki-cho, Nishinomiya, Hyogo 663-8558, Japan; Email: mw699032@mukogawa-u.ac.jp; 3 Research Institute, Marusan-Ai Co., Ltd., 1 Aza-Arashita, Nikki-cho, Okazaki, Aichi 444-2193, Japan; Email: Shintaro.egusa@marusanai.co.jp

**Keywords:** fermented soymilk, cholesterol, lipid metabolism

## Abstract

The effect of fermented soymilk on rats fed a high cholesterol diet was investigated to clarify the cholesterol-lowering function. Male Sprague-Dawley rats aged 7 weeks were fed a control diet (1% cholesterol, high cholesterol diet), high cholesterol diet containing 11.7% fermented soymilk diet (5% soy protein as final concentration, F-5), or high cholesterol diet containing 23.4% fermented soymilk diet (10% soy protein as final concentration, F-10) for 5 weeks. The liver weight and fat mass were decreased by the ingestion of fermented soymilk. The hepatic triglyceride and cholesterol levels in the F-5 and F-10 groups were significantly lowered compared to those in the control group. The plasma total cholesterol level of the F-10 group was significantly decreased. The expression of SREBP-2, a cholesterol synthesis-related gene, was significantly decreased in liver of the F-5 group, but the expression of CYP7a1, a cholesterol catabolism-related gene, was significantly increased. These results suggest that fermented soymilk can modulate the cholesterol metabolism in rats fed a high cholesterol diet.

## Abbreviations

CYP7a1: cytochrome p450 family 7 subfamily a polypeptide 1
FAS: fatty acid synthase
GAPDH: glyceraldehyde-3-phosphate dehydrogenase
LXRα: liver X receptor alpha
SREBP-1: sterol regulatory element binding protein 1
SREBP-2: sterol regulatory element binding protein 2

## 1. Introduction

Cardiovascular disease (CVD) is a severe problem in developed and developing countries. WHO warns that 17.3 million people died from CVD in 2008, and 23.6 million people a year will die from CVD by 2030. CVD is a disease mainly caused by atherosclerosis [1]. One of the risk factors of atherosclerosis is hypercholesterolemia [2,3] and low density lipoprotein cholesterol (LDL-C) is the major cause of onset of the atherogenic process [1] Soy foods, traditional food products in Asia [4], have been known to exhibit lipid metabolism-modulating effects since the 1970s. The major bioactive ingredients of soy foods are soy proteins and isoflavones. Much evidence on soy protein has been reported from studies on experimental animals and human subjects. Soy protein in the diet reduces the concentration of total cholesterol (TC) and LDL-C in plasma or serum [5,6,7,8]. Sugano *et al* found that the undigested insoluble fraction obtained by treating soy protein with microbial protease has a hypocholesterolemic effect in rats fed a cholesterol-enriched diet [8]. Soy protein also reduced the triglyceride (TG) concentrations in the plasma and liver in experimental animals and human [5,9,10,11,12]. Another bioactive ingredient, isoflavone, has also shown much lipid metabolism-modulating effects. Isoflavone lowered serum TC and LDL-C levels in a meta-analysis of 11 randomized controlled trials in humans [13]. In contrast, alcohol washed-soy protein, isoflavone-free form, decreased lowering of plasma TC level compared with intact soy protein on rodents [14]. Isoflavone extracted from soy protein decreased plasma TC level in rodents [14]. As soymilk has a lot of soy protein and isoflavone, the intake of soymilk is known to reduce plasma and hepatic lipid levels [15,16]. However, the taste of soymilk is not always favorable for everybody. As fermented soymilk prepared by *Bifidobacterium breve* YIT 4065 improved the lipid metabolism [17,18], we prepared fermented soymilk using lactic acid bacteria of vegetable origin. We found the effects of lactic acid-fermented soymilk containing okara on the plasma and hepatic lipid profiles and expression of the lipid metabolism-related genes in rats [19]. The administration of fermented soymilk lowered plasma cholesterol and TG levels compared with that of soymilk. Therefore, we assumed that some bioactive components produced from soymilk by lactic acid fermentation lowered plasma cholesterol and TG levels through modulating hepatic gene expression [20]. As a previous animal experiment was mainly investigated using rats fed AIN-93G diet [20], the effects of fermented soymilk on rats fed a normal diet were found. However, the effect of fermented soymilk on a high cholesterol diet is not yet resolved. Therefore, in the present study, we examined changes in plasma and hepatic cholesterol levels and analyzed the expression of lipid metabolism-related genes of the liver to clarify hypocholesterolemic effects of fermented soymilk on rats fed a high cholesterol diet.

## 2. Materials and Methods

### 2.1. Diets

The fermented soymilk was prepared from soymilk by lactic acid fermentation using *Lactobacillus delbrueckii* subsp. *delbrueckii* strain of TUA-4408L (SNC33) for 15 h and immediately freeze-dried for the animal experiments. The composition and energy of fermented soymilk are shown in Table 1. Total isoflavone content in aglycon equivalent was approximately 500 µmol/100 g of dried fermented soymilk. The other feed materials were purchased from Clea Japan (Tokyo, Japan), Nacalai Tesque (Kyoto, Japan) and Wako Pure Chemical Industries (Osaka, Japan).

**Table 1:** Composition and energy of freeze-dried fermented soymilk.

Component	Fermented soymilk	
	%	Energy (kcal/100 g)
Water	4.9	-
Protein	42.9	171.6
Fat	36.8	331.2
Carbohydrate	2.6	10.4
Dietary fiber	7.2	14.4
Minerals	5.6	-
Total energy	-	527.6

### 2.2. Animals and Feeding

Twenty four male Sprague-Dawley rats (7 weeks old) were purchased from Nihon SLC (Hamamatsu, Japan) and individually housed in cages at 23 ± 1 °C and a humidity of 55 ± 7% with a 12 h light-dark cycle. All the rats were acclimated on an AIN-93G diet [21] for 1 week to stabilize the metabolic conditions before the feeding experiments. The rats were assigned to three groups (*n* = 8) which did not exhibit any significant difference in the body weight and serum total cholesterol concentration from each other. The control (C) group was fed with the AIN-93G diet containing 1% cholesterol (control diet). The 5% soy protein containing-fermented soymilk (F-5) group was fed with a diet in which 11.7% of the control diet had been replaced with dried fermented soymilk, and the 10% soy protein containing-fermented soymilk (F-10) group was fed with a diet in which 23.4% of the control diet had been replaced with fermented soymilk (Table 2). These diets were prepared to equal amount of carbohydrate, protein, fat and dietary fiber, from each other. Thus, the diets were formulated to be isocaloric. The rats in each group were fed for 5 weeks and provided with permitted *ad libitum* access to the diet and water. The food intake and body weight were measured on alternate days. To measure the plasma parameters, all the rats were fasted for 8 h (8:00–16:00) on one day of each week, and blood were withdrawn by tail vein under anesthesia. The plasma immediately obtained by centrifugation was stored at −80 °C until needed. The plasma of fasted rats was used to measure TC, high density lipoprotein cholesterol (HDL-C) and TG levels. To examine the influence of diet on hepatic gene expression, non-fasted rats were sacrificed without any affliction under anesthesia after feeding period, and the liver was quickly removed and washed with ice-cold physiological saline. After the weights of tissues had been measured, the liver tissues were immediately immersed in RNA later (Qiagen, Tokyo, Japan) and kept at −80°C until needed. All animal experiments were performed according to the guidelines of the Animal Use Committee of Mukogawa Women’s University.

**Table 2:** Composition of the experimental diets.

Ingredient	Diet group			
	AIN-93G *	C	F-5	F-10
Casein ^1^ (%)	20.0	20.0	14.2	8.4
Cornstarch ^1^ (%)	39.8	38.8	38.1	36.5
Dextrinized cornstarch ^1^ (%)	13.2	13.2	13.0	12.5
Sucrose ^1^ (%)	10.0	10.0	10.0	10.0
Soybean oil ^1^ (%)	7.0	7.0	2.8	0.0
Cellulose ^1^ (%)	5.0	5.0	4.1	3.2
Mineral mix (AIN-93G-MX) ^1^ (%)	3.5	3.5	3.5	3.5
Vitamin mix (AIN-93-VX) ^1^ (%)	1.0	1.0	1.0	1.0
L-Cystine ^2^ (%)	0.3	0.3	0.3	0.3
Choline bitartrate ^3^ (%)	0.25	0.25	0.25	0.25
*tert*-Butylhydroquinone ^2^ (%)	0.0014	0.0014	0.0014	0.0014
Cholesterol ^2^ (%)	0.0	1.0	1.0	1.0
Fermented soymilk ^4^ (%)	0.0	0.0	11.7	23.4
Total (%)	100.0	100.0	100.0	100.0
Energy (kcal/100 g)	372.2	368.7	366.4	371.6

C, control; F-5, 11.7% of the control diet was replaced with dried fermented soymilk to 5% soy protein as final concentration; F-10, 23.4% of the control diet was replaced with fermented soymilk to 10% soy protein as final concentration; ^1^ Clea Japan, Osaka, Japan; ^2^ Wako Pure Chemical Industries, Osaka, Japan; ^3^ Nacalai Tesque, Kyoto, Japan; ^4^ Marusan-Ai, Okazaki, Japan; * AIN-93G diet [21].

### 2.3. Measurement of the Plasma and Liver Metabolic Parameters

Plasma TC, HDL-C and TG were enzymatically measured by using commercial kits (Cholesterol E-test, HDL-cholesterol E-test and Triglyceride E-test, Wako Pure Chemical Industries). The non-HDL-C concentration was calculated as (non-HDL-C) = (TC) − (HDL-C). The hepatic lipids were extracted by the ordinary method of Folch *et al.* [22]. The hepatic cholesterol and TG concentrations were enzymatically determined by using commercial kits (Cholesterol E-test and Triglyceride E-test, Wako Pure Chemical Industries, Osaka, Japan).

### 2.4. X-ray Computed Tomography (CT) Analysis

All the rats were subjected to X-ray computed tomography (CT) analysis using LaTheta LCT-200 (Aloka, Tokyo, Japan) under anesthesia at initial and end points of feeding period. Contiguous images at 3-mm slice between 12th breast bone and testes were used for quantitative assessment using LaTheta software (version 2.0) according to the manufacturer’s protocol. Densitometric calculations of fat tissues were performed using CT software (Aloka, Tokyo, Japan) using attenuation number thresholds of −500 to −140 for fat tissue. Visceral fat and subcutaneous fat were distinguished by CT value and evaluated quantitatively. The difference of each fat mass between initial and end points of feeding period was measured.

### 2.5. Real-Time Reverse Transcription-Polymerase Chain Reaction (RT-PCR)

The expression of mRNA was quantitatively measured by real-time RT-PCR, using the model 7500 (Applied Biosystems, Foster City, CA, USA) and related reagent kits according to the manufacturer’s protocol. Total RNA was extracted from liver tissues with the RNeasy mini kit (Qiagen). Complementary DNA (cDNA) was synthesized from the RNA using the Prime Script^®^ RT reagent Kit with gDNA Eraser (Perfect Real Time) (TAKARA BIO, Shiga, Japan). The following TaqMan gene expression assays were conducted using Rn01495769_ml for *Srebf1* (mRNA of SREBP-1), Rn00569117_ml for *Fasn *(mRNA of FAS), Rn00581185_ml for *Nr1h3* (mRNA of LXRα), Rn01502638_ml for *Srebf2* (mRNA of SREBP-2), and Rn00564065_ml for *Cyp7a1* (mRNA of CYP7a1) (Applied Biosystems) as the PCR primer set for the real-time PCR. Rn99999916_s1 for GAPDH was used as an endogenous control. Real-time PCR was performed with the TaqManUniversal PCR master mix (Applied Biosystems). Data were normalized to GAPDH RNA expression, and the fold was presented as a ratio to the C group.

### 2.6. Statistical Analysis

The results are presented as the mean ± standard error. Tukey’s multiple comparison was used for blood analysis at each feeding time, hepatic lipid parameters and fat mass increased between initial and end points of feeding period. Student’s *t*-test was used for the real time RT-PCR data of hepatic gene expression. The statistical analyses were performed with SPSS 12.0 J for Windows.

## 3. Results

### 3.1. Body Weight, Food Intake, Food Efficiency, Total Energy Intake and Tissue Weights

No significant difference in final body weight, food intake, food efficiency and total energy intake was found among the three groups (Table 3). The liver weight of the F-10 group after 5 weeks was significantly decreased compared with that of the C group. The cecum weight of the F-10 group was significantly increased compared with those of the C and F-5 groups.

**Table 3:** Initial and final body weights, food intake, food efficiency, total energy intake and tissue weights of rats fed on the experimental diets for 5 weeks.

	C	F-5	F-10
Initial body weight (g)	271.7 ± 3.5 ^a^	269.5 ± 3.4 ^a^	266.2 ± 2.9 ^a^
Final body weight (g)	422.1 ± 11.6 ^a^	401.1 ± 15.2 ^a^	394.9 ± 13.0 ^a^
Food intake (g/day)	19.8 ± 0.7 ^a^	19.1 ± 0.7 ^a^	18.3 ± 0.7 ^a^
Food efficiency			
(g b.w. gain/g diet)	0.23 ± 0.01 ^a^	0.20 ± 0.02 ^a^	0.21 ± 0.01 ^a^
Total energy uptake (kcal)	2769.7 ± 96.3 ^a^	2651.9 ± 99.1 ^a^	2562.4 ± 97.2 ^a^
Tissue weight (% b.w.)			
Liver	4.6 ± 0.1 ^a^	4.1 ± 0.1 ^ab^	3.6 ± 0.1 ^b^
Cecum	1.0 ± 0.1^a^	1.0 ± 0.1 ^a^	1.2 ± 0.1 ^b^

Each value is the mean ± SE for 8 rats. ^a,b^ Means not sharing a common superscript differ significantly by Tukey’s multiple-comparison test (*p* < 0.05).

### 3.2. Visceral Fat and Subcutaneous Fat Masses

**Figure 1:**
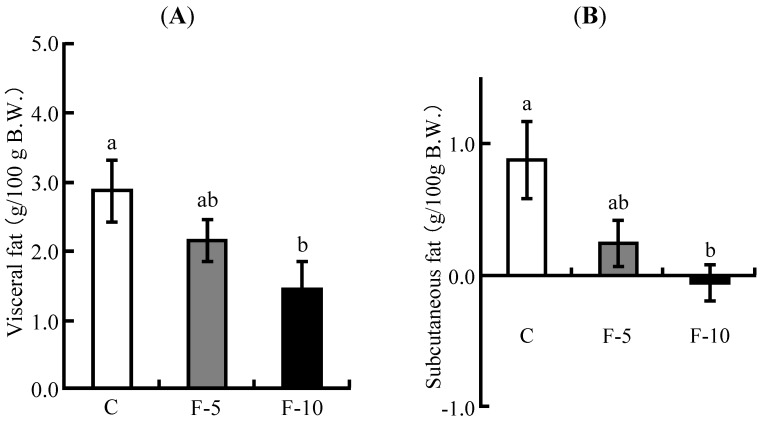
Comparison of increased amount of visceral and subcutaneous fat masses in the three groups. (**A**) visceral fat; (**B**) subcutaneous fat. The difference of each fat mass between the initial and end points of feeding period was measured. These fat mass regions were estimated by X-ray CT scan. Each value is the mean ± SE of 8 rats. ^a,b^ Means not sharing a common superscript differ significantly by Tukey’s multiple-comparison test (*p* < 0.05).

All the rats were subjected to X-ray CT analysis to calculate visceral and subcutaneous fat masses at initial and end points of the feeding period. Increased mass of each fat tissue between the initial and end points was calculated. The F-5 and F-10 groups suppressed the increased amount of visceral fat mass (Figure 1A). Especially, the F-10 group significantly reduced the amount compared with the C groups. Similarly, the increased mass of subcutaneous fat was significantly suppressed in the F-10 group (Figure 1B).

### 3.3. Hepatic Lipid Profile

**Figure 2:**
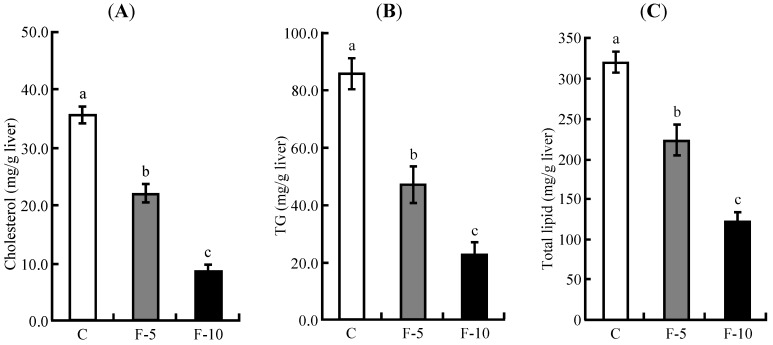
Liver parameters of rats fed on the experimental diets for 5 weeks. (**A**) hepatic cholesterol; (**B**) hepatic TG; (**C**) hepatic total lipid. Each value is the mean ± SE for 8 rats. ^a,b,c^ Means not sharing a common superscript differ significantly by Tukey’s multiple-comparison test (*p* < 0.05).

Hepatic cholesterol and TG levels were higher with a high cholesterol diet compared with those on cholesterol-free diet ingestion in our previous paper [20] and were increased to 13.7-fold and 4.6-fold *vs.* those on a cholesterol-free diet, respectively. The hepatic cholesterol, TG and total lipid levels in the F-5 and F-10 groups were significantly decreased in a dose dependent manner *vs*. those of the C group, respectively (Figure 2A,B,C). Furthermore, the hepatic cholesterol, TG and total lipid levels of the F-10 group were significantly decreased compared with those of the F-5 group.

### 3.4. Plasma Lipid Profiles

**Figure 3:**
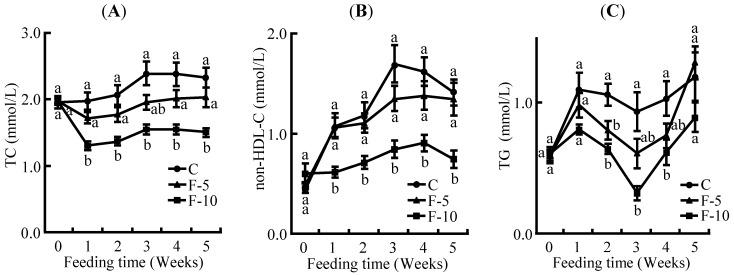
Plasma parameters of rats fed on the experimental diets for 5 weeks. (**A**) plasma TC level; (**B**) non-HDL-C; (**C**) plasma TG level. Each value is the mean ± SE for 8 rats. ^a,b ^Means not sharing a common superscript differ significantly by Tukey’s multiple-comparison test (*p* < 0.05).

Plasma TC and non-HDL-C levels in the C group fed a high cholesterol diet were higher compared with those on a cholesterol-free diet in our previous paper [20]. Plasma TG level was slightly lowered by a high cholesterol diet. Plasma TC level was decreased in a dose dependent manner by ingestion of fermented soymilk (Figure 3A). The F-10 group showed a significantly lower TC level from 1 week to 5 weeks. As shown in Figure 3B, plasma non-HDL-C was temporally decreased in a dose dependent manner by ingestion of fermented soymilk from 3 weeks to 4 weeks. The F-10 group displayed significantly decreased non-HDL-C compared with the C group from 1 week to 5 weeks. The HDL-C/TC ratio was significantly increased in the F-10 group compared with the C group (data not shown). Plasma TG level showed temporally significant decrease in the F-10 group from 2 weeks to 4 weeks (Figure 3C).

### 3.5. Real Time PCR Analysis

**Figure 4:**
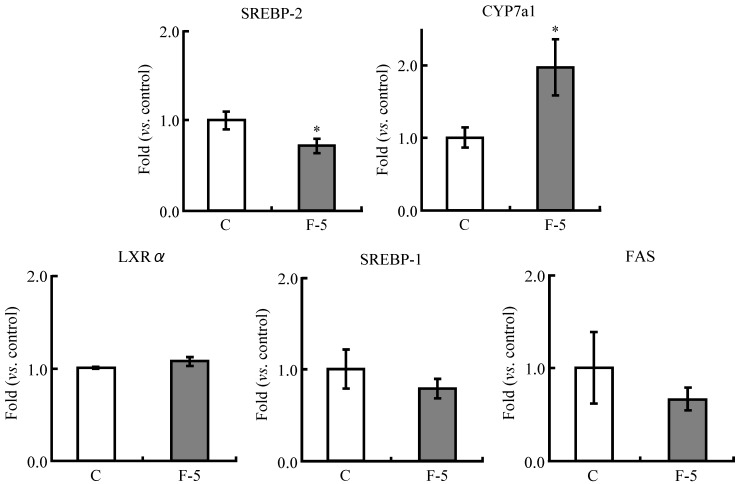
Expression of lipid metabolism-related genes in the liver of rats fed experimental diets for 5 weeks. The expression of mRNA was quantitatively measured by real-time RT-PCR. Each value is the mean ± SE for 8 rats. The data were normalized to GAPDH RNA expression and are presented as a ratio to the C value. Statistically significant compared with the control group (* *p* < 0.05; Student’s *t*-test).

With regard to the change of hepatic lipid profile by the ingestion of fermented soymilk, the gene expression of hepatic lipid metabolism in the only F-5 group was compared with those of the C group because the gene expression from livers of the F-10 group was not obtained (Figure 4). The gene expression of LXRα was scarcely changed in the F-5 group (1.1 fold). In cholesterol metabolism, the expression of CYP7a1 of the F-5 group was significantly up-regulated to 2.0 fold compared with that of the C group. In contrast, the expression of SREBP-2 was significantly decreased to 0.7 fold in the F-5 group compared with the C group. Although the gene expressions of SREBP-1 and FAS in fatty acid synthesis-related metabolism were down-regulated 0.8 and 0.7 fold, respectively, significant change of those expressions was not found in the F-5 group.

## 4. Discussion

The effects of lactic acid-fermented soymilk on lipid metabolism in rats fed an AIN-93G diet have been known [19,20]. In our previous paper [23], it was found that fermented soymilk diet containing 10% soy protein as final concentration was necessary to significantly enhance the lipid metabolism modulation in rats fed a cholesterol-free diet. Thus, in the present study, we examined the dose response of the lipid metabolism-modulation effect by using different concentrations of fermented soymilk in rats fed a high cholesterol diet. Furthermore, the effect of lipid metabolism modulation on rats fed a high cholesterol diet was compared with that on rats fed a cholesterol-free diet in our previous paper [20].

The consumption of fermented soymilk resulted in reduction of liver weight (Table 3) and decreased hepatic cholesterol and TG levels in a dose dependent manner (Figure 2A,B). The hepatic total lipid level was also decreased in a dose dependent manner in the presence of fermented soymilk (Figure 2C). Although the C group fed a high cholesterol diet showed higher levels of hepatic cholesterol, TG and total lipid compared with those of the AIN-93G diet group in our previous paper [20], the levels in the F-5 and F-10 groups were decreased by fermented soymilk intake. Especially, hepatic total lipid and TG levels in the F-10 group were decreased to the same ones as those of rats fed an AIN-93G diet [20] respectively, and the hepatic cholesterol level in the F-10 group was decreased to lower one than that of fed an AIN-93G diet [20]. These data indicated that the reduction of hepatic cholesterol and TG levels by fermented soymilk intake leads to prevention of hepatic lipid accumulation or fatty liver. Moreover, the increased amounts of visceral and subcutaneous fat masses were suppressed in the fermented soymilk group (Figure 1A,B). Especially, the fat mass increase in the F-10 group was significantly reduced. Such a suppression of fat mass increase seems to be caused by lowering hepatic lipid and plasma TG levels, because plasma TG level in the F-10 group was continuously decreased from 1 week to 5 weeks (Figure 3C). In contrast, plasma TC level increased by high cholesterol diet was decreased in the dose dependent manner of fermented soymilk. Although the lowering of plasma TC level in the F-5 group did not show a significant difference, the TC level was decreased to the same one as that of the AIN-93G diet group [20]. The TC level in the F-10 group showed lower one than that of the AIN-93G diet group. Additionally, although LDL-C was directly measured, the ingestion of fermented soymilk inhibited the increase of plasma non-HDL-C as shown in the F-10 group (Figure 3B) and the level was the same as that of the AIN-93G diet group [20]. LDL-C is the major cause of onset of atherosclerosis [1]. Therefore, results of this study suggest that the consumption of fermented soymilk may reduce atherosclerosis. 

The hepatic cholesterol and TG levels were affected by ingestion of fermented soymilk diet containing more than 5% soy protein as shown in Figure 2. In contrast, the fermented soymilk diet containing 10% soy protein was essential to obtain the significant reduction of plasma TC level, non-HDL-C level and adipose tissue mass (Figure 1 and Figure 3). Although 20% soy protein in the diet has been often used to investigate the improvement of lipid metabolism in rats [24,25,26], the soy protein concentration of fermented soymilk diets used in the present study was 5% or 10%. It was assumed that a relatively low concentration of soy protein in fermented soymilk diet exhibits a hypocholesterolemic effect. Lowering of cholesterol levels have been reported by using functional substances such as soy protein [27], soy peptide [28,29], isoflavone [30] and saponin [31]. As soymilk includes many kinds of functional substances, cholesterol-lowering effects of fermented soymilk may be caused by not only soy protein or peptide but also by isoflavone and saponin. 

Although the combination of plant sterol ester with soy protein increased the fecal excretion of bile acid [9], in the present study, the significant difference in bile acid level of feces was not found between the control group and the fermented soymilk groups. However, as the administration of fermented soymilk diet containing 5% soy protein significantly affected hepatic lipid profiles in rats, the gene expressions of lipid metabolisms in the F-5 group were examined compared with those of the C group (Figure 4). The gene expression of LXRα was scarcely changed in the F-5 group, and the changes in expressions of SREBP-1 and FAS [32], the fatty acid synthesis-related genes controlled by LXRα, were also not statistically significant (Figure 4). In contrast, CYP7a1, the rate-limiting enzyme in the formation of bile acid from cholesterol [33] controlled by LXRα [34], was increased as shown in the fermented soymilk group fed a cholesterol-free diet in our previous paper [20]. In the present study, in spite of almost invariable expression of LXRα, the expression of CYP7a1 was significantly up-regulated in the F-5 group (Figure 4). Therefore, it seems that other gene than LXRα strongly controlled the expression of CYP7a1. Farnesoid X receptor (FXR) and pregnane X receptor (PXR) is known to inhibit CYP7a1 [34]. The enhancement of CYP7a1 expression was likely to be involved in suppressing FXR and PXR. In addition, the expression of SREBP-2, cholesterol synthesis-accelerating factor [35], was decreased in the F-5 group. In the present study, protein levels of SREBP-2 and CYP7a1 were not measured. However, if the down-regulation of SREBP-2 and up-regulation of CYP7a1 reflect the amount of enzymes related to cholesterol metabolism, the fermented soymilk might decrease the excess pool of cholesterol incorporated through a high cholesterol diet. We must examine the abundance of SREBP-2 and CYP7a1 proteins to confirm changes of cholesterol synthesis and catabolism.

## 5. Conclusions

Fermented soymilk prevented hypercholesterolemia by modulating cholesterol metabolism. Therefore, the consumption of fermented soymilk seems to be available for CVD prevention.

## Implication

This study shows the potential importance of fermented foods for prevention of metabolic syndrome.

## References

[1] Napoli C., Crudele V., Soricelli A., Al-Omran M., Vitale N., Infante T., Mancini F.P. (2012). Primary prevention of atherosclerosis: A clinical challenge for the reversal of epigenetic mechanisms?. Circulation.

[2] Kannel W.B., Castelli W.P., Gordon T., McNamara P.M. (1971). Serum cholesterol, lipoproteins, and the risk of coronary heart disease. Ann. Intern. Med..

[3] Ross R., Harker L. (1976). Hyperlipidemia and atherosclerosis. Science.

[4] Golbitz P. (1995). Traditional soyfoods: Processing and products. J. Nutr..

[5] Carroll K.K. (1991). Review of clinical studies on cholesterol-lowering response to soy protein. J. Am. Diet. Assoc..

[6] Kritchevsky D. (1993). Dietary protein and experimental atherosclerosis. Ann. N. Y. Acad. Sci..

[7] Sirtori C.R., Lovati M.R., Manzoni C., Monetti M., Pazzucconi F., Gatti E. (1995). Soy and cholesterol reduction: Clinical experience. J. Nutr..

[8] Sugano M., Koba K. (1993). Dietary protein and lipid metabolism: A multifunctional effect. Ann. N. Y. Acad. Sci..

[9] Lin Y., Meijer G.W., Vermeer M.A., Trautwein E.A. (2004). Soy protein enhances the cholesterol-owering effect of plant sterol esters in cholesterol-fed hamsters. J. Nutr..

[10] Moriyama T., Kishimoto K., Nagai K., Urade R., Ogawa T., Utsumi S., Maruyama N., Maebuchi M. (2004). Soybean beta-conglycinin diet suppresses serum triglyceride levels in normal and genetically obese mice by induction of beta-oxidation, downregulation of fatty acid synthase, and inhibition of triglyceride absorption. Biosci. Biotechnol. Biochem..

[11] Ascencio C., Torres N., Isoard-Acosta F., Gómez-Pérez F.J., Hernández-Pando R., Tovar A.R. (2004). Soy protein affects serum insulin and hepatic SREBP-1 mRNA and reduces fatty liver in rats. J. Nutr..

[12] Wang Y., Jones P.J., Ausman L.M., Lichtenstein A.H. (2004). Soy protein reduces triglyceride levels and triglyceride fatty acid fractional synthesis rate in hypercholesterolemic subjects. Atherosclerosis.

[13] Taku K., Umegaki K., Sato Y., Taki Y., Endoh K., Watanabe S. (2007). Soy isoflavones lower serum total and LDL cholesterol in humans: A meta-analysis of 11 randomized controlled trials. Am. J. Clin. Nutr..

[14] Demonty I., Lamarch B., Jones P.J. (2003). Role of isoflavones in the hypocholesterolemic effect of soy. Nutr. Rev..

[15] Chen J.R., Liu S.M., Yang S.C., Suetsuna K. (2004). Soymilk intake is associated with plasma and liver lipid profiles in rats fed a high-cholesterol diet. Nutrition.

[16] Choi J.Y., Jeon J.E., Jang S.Y., Jeong Y.J., Jeon S.M., Park H.J., Choi M.S. (2011). Differential effects of powdered whole soy milk and its hydrolysate on antiobesity and antihyperlipidemic response to high-fat treatment in C57BL/6N mice. J. Agric. Food. Chem..

[17] Kikuchi-Hayakawa H., Onodera N., Matsubara S., Yasuda E., Chonan O., Takahashi R., Ishikawa F. (1998). Effects of soy milk and bifidobacterium fermented soy milk on lipid metabolism in aged ovariectomized rats. Biosci. Biotechnol. Biochem..

[18] Kikuchi-Hayakawa H., Onodera N., Matsubara S., Yasuda E., Shimakawa Y., Ishikawa F. (1998). Effects of soya milk and Bifidobacterium-fermented soya milk on plasma and liver lipids, and faecal steroids in hamsters fed on a cholesterol-free or cholesterol-enriched diet. Br. J. Nutr..

[19] Kitawaki R., Nishimura Y., Takagi N., Iwasaki M., Tsuzuki K., Fukuda M. (2009). Effects of *Lactobacillus* fermented soymilk and soy yogurt on hepatic lipid accumulation in rats fed a cholesterol-free diet. Biosci. Biotechnol. Biochem..

[20] Kobayashi M., Harada T., Takagi N., Tsuzuki K., Sugawara M., Fukuda M. (2012). Effects of lactic acid-fermented soymilk on lipid metabolism-related gene expression in rat liver. Biosci. Biotechnol. Biochem..

[21] Reeves P.G., Nielsen F.H., Fahey G.C. (1993). AIN-93 purified diets for laboratory rodents: Final report of the American Institute of Nutrition ad hoc writing committee on the reformulation of the AIN-76A rodent diet. J. Nutr..

[22] Folch J., Lees M., Sloane Stanley G.H. (1957). A simple method for the isolation and purification of total lipids from animal tissues. J. Biol. Chem..

[23] Harada T., Tanaka M., Tsuzuki K., Sugawara M., Takagi N., Fukuda M. (2010). Effective Dosage of Lactic Fermented Soymilk for Improving Lipid Metabolism in Rats (in Japanese). Nippon Shokuhin Kagaku Kogaku Kaishi.

[24] Tovar A.R., Murguia F., Cruz C., Hernandez-Pando R., Aguilar S.C.A., Pedraza-Chaverri J., Correa-Rotter R., Torres N. (2002). A soy protein diet alters hepatic lipid metabolism gene expression and reduces serum lipids and renal fibrogenic cytokines in rats with chronic nephrotic syndrome. J. Nutr..

[25] Torres N., Torre-Villalvazo I., Tovar A.R. (2006). Regulation of lipid metabolism by soy protein and its implication in diseases mediated by lipid disorders. J. Nutr. Biochem..

[26] Simmen F.A., Mercado C.P., Zavacki A.M., Huang S.A., Greenway A.D., Kang P., Bowman M.T., Prior R.L. (2010). Soy protein diet alters expression of hepatic genes regulating fatty acid and thyroid hormone metabolism in the male rat. J. Nutr. Biochem..

[27] Nagata Y., Tanaka K., Sugano M. (1981). Further studies on the hypocholesterolaemic effect ofsoya-bean protein in rats. Br. J. Nutr..

[28] Nagaoka S., Awano T., Nagata N., Masaoka M., Hori G., Hashimoto K. (1997). Serum cholesterol reduction and cholesterol absorption inhibition in Caco-2 cells by a soy protein peptic hydrolyze. Biosci. Biotechnol. Biochem..

[29] Nagaoka S., Miwa K., Eto M., Kuzuya Y., Hori G. (1999). Soy protein peptic hydrolysate with bound phospholipids decreases micellar solubility and cholesterol absorption in rats and Caco-2 cells. J. Nutr..

[30] Lucas E.A., Khalil D.A., Daggy B.P., Arjmandi B.H. (2001). Ethanol-extracted soy protein isolate does not modulate serum cholesterol in Golden Syrian hamsters: A model of postmenopausal hypercholesterolemia. J. Nutr..

[31] Han L.K., Zheng Y.N., Xu B.J., Okuda H., Kimura Y. (2002). Saponins from platycodi radix ameliorate high fat diet-induced obesity in mice. J. Nutr..

[32] Joseph S.B., Laffitte B.A., Patel P.H., Watson M.A., Matsukuma K.E., Walczak R., Collins J.L., Osborne T.F., Tontonoz P. (2002). Direct and indirect mechanisms for regulation of fatty acid synthase gene expression by liver X receptors. J. Biol. Chem..

[33] Myant N.B., Mitropoulos K.A. (1977). Cholesterol 7 alpha-hydroxylase. J. Lipid Res..

[34] Chen J., Raymond K. (2006). Nuclear receptors, bile-acid detoxification, and cholestasis. Lancet.

[35] Horton J.D., Shimomura I., Brown M.S., Hammer R.E., Goldstein J.L., Shimano H. (1998). Activation of cholesterol synthesis in preference to fatty acid synthesis in liver and adipose tissue of transgenic mice overproducing sterol regulatory element-binding protein-2. J. Clin. Invest..

